# Biotin-Streptavidin Competition Mediates Sensitive Detection of Biomolecules in Enzyme Linked Immunosorbent Assay

**DOI:** 10.1371/journal.pone.0151153

**Published:** 2016-03-08

**Authors:** Thangavel Lakshmipriya, Subash C. B. Gopinath, Thean-Hock Tang

**Affiliations:** 1 Advanced Medical & Dental Institute (AMDI), Universiti Sains Malaysia, Kepala Batas, Penang, Malaysia; 2 Institute of Nano Electronic Engineering (INEE), Universiti Malaysia Perlis, Kangar, Perlis, Malaysia; 3 School of Bioprocess Engineering, Universiti Malaysia Perlis, Arau, Perlis, Malaysia; CNR, ITALY

## Abstract

Enzyme Linked Immunosorbent Assay (ELISA) is the gold standard assay for detecting and identifying biomolecules using antibodies as the probe. Improving ELISA is crucial for detecting disease-causing agents and facilitating diagnosis at the early stages of disease. Biotinylated antibody and streptavidin-conjugated horse radish peroxide (streptavidin-HRP) often are used with ELISA to enhance the detection of various kinds of targets. In the present study, we used a competition-based strategy in which we pre-mixed free biotin with streptavidin-HRP to generate high-performance system, as free biotin occupies some of the biotin binding sites on streptavidin, thereby providing more chances for streptavidin-HRP to bind with biotinylated antibody. ESAT-6, which is a protein secreted early during tuberculosis infection, was used as the model target. We found that 8 fM of free biotin mixed with streptavidin-HRP anchored the higher detection level of ESAT-6 by four-fold compared with detection without free biotin (only streptavidin-HRP), and the limit of detection of the new method was 250 pM. These results suggest that biotin-streptavidin competition can be used to improve the diagnosis of analytes in other types of sensors.

## Introduction

Characteristics of desired analyte and ligand molecules, which include enzymes, proteins, antibodies, nucleic acids, and glycans, are the primary criteria to be considered when designing sensing mechanisms. Measurement of the signal generated upon analyte and ligand interaction is the basis of such sensing devices [1–7]. This approach has been applied to disease diagnosis, environmental monitoring, drug discovery, drug screening, therapeutics, and extension of the human life span [8–15]. A good biosensing system must have both high sensitivity and selectivity. A system that can detect low levels of an analyte in crude samples such as serum or urine is crucial for identifying diseases at the early stage, which is important because treatment and control are easier when the disease is caught early. In addition, an effective system requires use of the right molecules or biomarkers to detect a given disease [4,16–20]. Various immunoassays with high sensitivity have been developed to diagnose the presence of analyte molecules using antibodies as the probe [21–25]. Among these, Enzyme Linked Immunosorbent Assay (ELISA) is one of the most efficient methods available to identify disease-causing agents [26–31]. ELISA is an easy-to-use, sensitive, high-throughput method that requires only a simple equipment [21,32,33].

The ELISA method can be improved to facilitate better level of detections and to be adaptable to a wide range of applications. For example, researchers have used different approaches, such as molecular complementation, to improve the limit of detection (LOD) of ELISA [33]. Sensitivity of ELISA depends on factors such as binding strength of biomolecules, surface functionalization, and molecular assembly. In particular, the detection limit of the system depends greatly on the number of capturing molecules bound to the ELISA surface. Molecular capturing and immobilization vary with different conditions, including pH, temperature, and charge on the sensor surface and protein [34]. Thus, the use of modified surface molecules with proper orientation of the analyte and capturing molecules can improve the sensitivity of the system. Biomolecules are immobilized on the ELISA plate mainly through chemical, physical, or electrostatic interaction. The ELISA plate is made of polystyrene (PS), so the antibody or protein generally is immobilized through the COOH-link on the PS. However, it is difficult to immobilize small molecules in this manner.

Vashist et al. (2014) [34] developed a method of one-step immobilization of antibody on the ELISA plate and showed that it enhanced the detection limit of the system. Nanoparticle-conjugated antibody or protein have also been shown to improve the LOD of ELISA; in particular, antibody-conjugated gold nanoparticles (GNPs) were found to improve the system’s sensitivity [35]. Similarly, the biotin-streptavidin conjugation strategy is commonly used in ELISA protocols to increase the LOD. Biotin-streptavidin is a powerful non-covalent interaction with high affinity and a dissociation constant of 2.3 x 10^13^ M^–1^ [36]. Each streptavidin molecule has four binding sites for biotin, and these binding opportunities are useful in different biological applications. Streptavidin also can be tagged with biomolecules such as enzymes, antibodies, or GNPs to improve detection of the system. The biotin-streptavidin interaction has been used in many biological applications, including sensor development, bio-imaging, drug delivery, and protein purification.

In this study, we utilized the biotin-streptavidin interaction with ELISA to include a competition-based strategy for enhancing the detection. Horseradish peroxide (HRP) conjugated streptavidin (streptavidin-HRP) was used to detect the analyte in the final step by reacting the substrate for HRP. Due to high-affinity between biotin and streptavidin, there will be a good sensitivity. Further, to enhance the sensitivity, we made competition between biotinylated antibody and streptravidin-HRP by externally adding free biotin. The improvement in sensitivity of this competition is due to four binding sites for biotin on streptavidin. More importantly, we pre-mixed free biotin with streptavidin-HRP prior to the experiments. Free biotin blocks some of the binding sites for biotin on the streptavidin-HRP molecules, thus providing more chances for streptavidin-HRP to bind with the biotinylated secondary antibody already immobilized on the ELISA plate. After immobilize the biotinylated antibody on the ELISA plate, we introduced this pre-mix and promoted the sensitivity. We used ESAT-6 (molecular weight 6 kDa [37]), a protein secreted early during tuberculosis infection, as the model protein. We postulated that this approach would be able to detect the presence of low levels of the target.

## Materials and Methods

### Reagents and biomolecules

ESAT-6 was purchased from Sino Biological Inc. (Beijing, China). Anti-ESAT-6 was obtained from Santa Cruz Biotechnology (USA), biotinylated anti-mouse-IgG was from Invitrogen (USA), streptavidin-HRP was procured from Thermo-Scientific (Japan), bovine serum albumin (BSA) was from Promega (USA), and. 16 kDa and Ag85B proteins were obtained from CalBioreagents (USA) and abcan (USA), respectively. ELISA coating buffer (5x) was from Biolegend (Japan). It composed of 0.1 M sodium carbonate (7.13 g NaHCO_3_, 1.59 g Na_2_CO_3_ in 1.0 L; pH 9.5). Substrate for HRP was purchased from Promega and biotin was obtained from Thermo Fisher Scientific, Malaysia. ELISA plates were purchased from Becton Dickinson (France), and the ELISA reader and Tween-20 were from R & M Chemicals (U.K.).

### Optimization of the biotinylated anti-mouse-IgG and streptavidin-HRP interaction

To determine the optimal dilution of biotinylated anti-mouse-IgG (biotinylated antibody) and streptavidin-HRP, ELISA plates were directly coated with biotinylated antibody (1 mg/mL) at a dilution of 1:1000 or 1:500 in 1X coating buffer. Each plate was incubated for 3 h at room temperature (RT). The remaining surfaces were blocked with 2% BSA for 1 h at RT, and then three different concentrations of streptavidin-HRP [from the stock; 2 mg/mL, diluted at 1:1000 (1 μg/mL), 1:5000 (200 ng/mL), and 1:10,000 (100 ng/mL)] were added and allowed to incubate for 1 h at RT. The wells were washed five times with the washing buffer (PBS containing 0.05% of Tween-20) between each step. Finally, the substrate for HRP was added to detect the interaction between biotin and streptavidin. The optical density (OD) measurement was done using the ELISA reader at the wavelength of 405 nm. Photographs were taken 10 min after adding the HRP substrate.

### Improvement of the biotin-streptavidin-based ELISA by the addition of free biotin

To evaluate whether the addition of free biotin improves the biotin-streptavidin assay, the optimized dilution (1:500) of biotinylated antibody (diluted in the 1X coating buffer) was coated onto the ELISA plate for 3 h at RT. The remaining binding places on the wells then were blocked with 2% BSA for 1 h. Different concentrations of free biotin (0 to 60 fM) were pre-mixed with the streptavidin-HRP (1:1000) and kept separately for 30 min. The molar ratios between free biotin and biotinylated antibody were calculated as 1:0.4x10^6^ for 0.25 fM (lowest concentration) and 1:8x10^7^ for 60 fM (highest concentration) of free biotin. The pre-mixtures of biotin and streptavidin-HRP then were added to the biotinylated antibody immobilized ELISA plate and allowed to incubate for 1 h. The wells were washed five times with washing buffer between each step. Finally, the substrate for HRP was added to detect the interaction between biotin and streptavidin. All experimental steps were performed at RT. The OD was read using the ELISA reader at 405 nm. Photographs were taken 10 min after adding the HRP substrate.

### Optimization of the interaction between anti-ESAT- 6 and ESAT-6

To determine the optimal dilution of anti-ESAT-6, 200 nM of ESAT- 6 were diluted in 1X coating buffer, coated onto the ELISA plate, and allowed to incubate for 3 h at RT. Next, 2% BSA was added to each well to cover the remaining binding places on the wells for 1 h. Three different dilutions of anti-ESAT-6 [from the stock; 1 mg/mL, diluted at 1:5000 (200 ng/mL), 1:1000 (1 μg/mL), and 1:500 (2 μg/mL)] then were added to the wells. The optimized concentration of biotinylated antibody (1:500) was added to each well and allowed to incubate for 1 h, and then the 1:1000 dilution of streptavidin-HRP was added to each well. A control experiment was performed without ESAT-6. Wells were washed five times with the washing buffer between each step. Finally, the substrate for HRP was added to detect the interaction between ESAT-6 and anti-ESAT-6. All experimental steps were performed at RT. The OD was read using the ELISA reader at 405 nm. Photographs were taken 10 min after adding the HRP substrate.

### Improvement in detection of ESAT-6 by free biotin with streptavidin-HRP

To confirm that the addition of free biotin to the system improves detection, ESAT-6 (100 nM) was diluted in the 1X coating buffer coated onto the ELISA plate and allowed to incubate for 3 h at RT. The ELISA wells were blocked with 2% BSA for 1 h. The optimized dilution of anti-ESAT-6 (1:500) then was added, followed by the optimized dilution of biotinylated antibody (1:500) and then finally the 1:1000 dilution of streptavidin-HRP pre-mixed with different concentrations of free biotin (streptavidin-HRP incubated with 0 to 60 fM of free biotin for 10 min). A control experiment was performed without ESAT-6. Wells were washed five times with washing buffer between each step. Finally, the substrate for HRP was added to detect the interaction between ESAT-6 and anti-ESAT-6. All experimental steps were performed at RT. The OD was read using the ELISA reader at 405 nm. Photographs were taken 10 min after adding the HRP substrate.

### Detection limit: Comparison between ELISA with and without free biotin

To determine the LOD of ESAT-6, ESAT-6 was titrated from 0.1 to 120 nM. The steps were as follows: (a) ESAT-6 was coated onto the ELISA plate; (b) 2% BSA blocking was performed; (c) 1:500 dilution of anti-ESAT-6 was added; (d) 1:500 dilution of biotinylated antibody was added; (e) 1:1000 dilution of streptavidin-HRP with and without free biotin was added; and (f) substrate for HRP was added. Wells were washed five times with the washing buffer between each step. Finally, the substrate for HRP was added to detect the binding of ESAT-6 on the ELISA well. All experimental steps were performed at RT. The OD was read using the ELISA reader at 405 nm. Photographs were taken 10 min after adding the HRP substrate.

### Specificity: Spiking human serum with ESAT-6

Specific detection of biomolecules in the crude samples such as serum is important to identify the particular target. To demonstrate the specificity, ESAT-6 was spiked into the human serum and detected the ESAT-6. This experiment will reveal the genuine detection with serum containing high concentration of BSA, Globulin and other proteins. The specificity of different concentrations of ESAT-6 (0 to 120 nM) spiked into a 1:1000 dilution of human serum was measured. The mixed sample was coated onto the ELISA plate and the other procedures were the same as those described above. To confirm the specific detection of ESAT-6, 250 pM of ESAT-6 was also spiked into human saliva, urine and with the mixture of other TB proteins (16 kDa, Ag85B), and detected as described above.

## Results and Discussion

ELISA is the gold standard method for detecting a given analyte molecule using a suitable antibody. Biotinylated secondary antibody and streptavidin-HRP are commonly used in the ELISA to improve the limit of detection (LOD). Streptavidin is a tetrameric protein with a molecular weight of 60 kDa; it has a high binding affinity to biotin (in the low femtomolar range) and four biotin binding sites [36]. Because of these properties, the biotin-streptavidin assay is commonly used in many biotechnological applications, including imaging [38], purification [39], drug delivery [40], and bioanalytical immunoassays [23,41].

**Fig 1:**
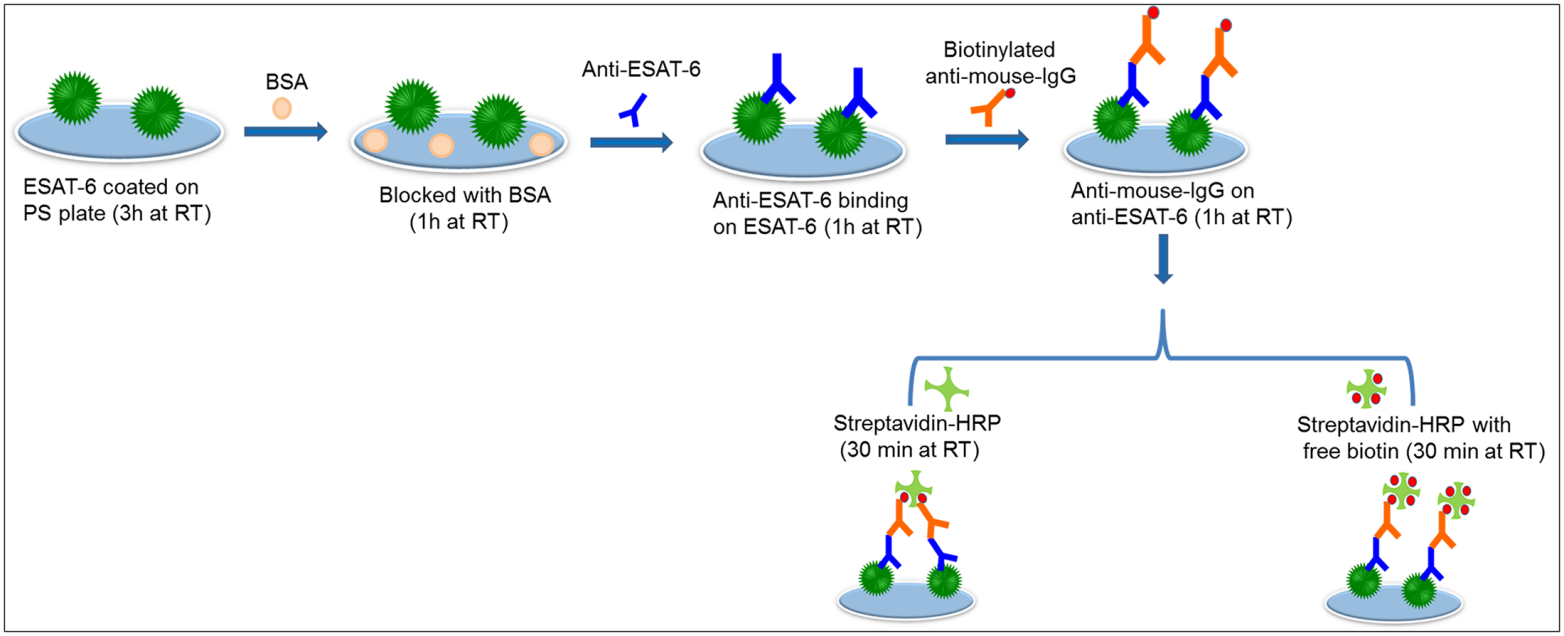
Schematic representation of ESAT-6 detection by the biotin-streptavidin interaction. Complete schematics are displayed. ESAT-6 protein was coated onto the plate followed by anti-ESAT-6 and biotinylated anti-mouse-IgG. Streptavidin-HRP with and without free biotin then was added.

The interaction between biotin and streptavidin can be further utilized to improve biomolecule detection by ELISA. Researchers have experimented with modifying the structure of streptavidin, especially the biotin binding sites, to increase the binding of biotin and streptavidin [42–44]. The four binding sites can be occupied completely by biotin immobilized on the sensing surface, leading to comparatively less binding than without free biotin [9]. In this study we blocked some of the biotin binding sites on streptavidin-HRP using free biotin in order to provide more chances for streptavidin-HRP to bind with biotinylated antibody. We found that free biotin pre-mixed with streptavidin-HRP improved the detection of ESAT-6. Fig 1 shows a schematic representation of the improved detection of ESAT-6 with the ELISA system modified by the addition of free biotin. After the interaction of anti-ESAT-6 and biotinylated anti-mouse-IgG, we introduced the mixture of streptavidin-HRP and free biotin, it gives more chance of streptavidin-HRP binding on the biotinylated anti-mouse-IgG, leads enhanced detection.

### Interaction between biotinylated anti-mouse-IgG and streptavidin-HRP

**Fig 2:**
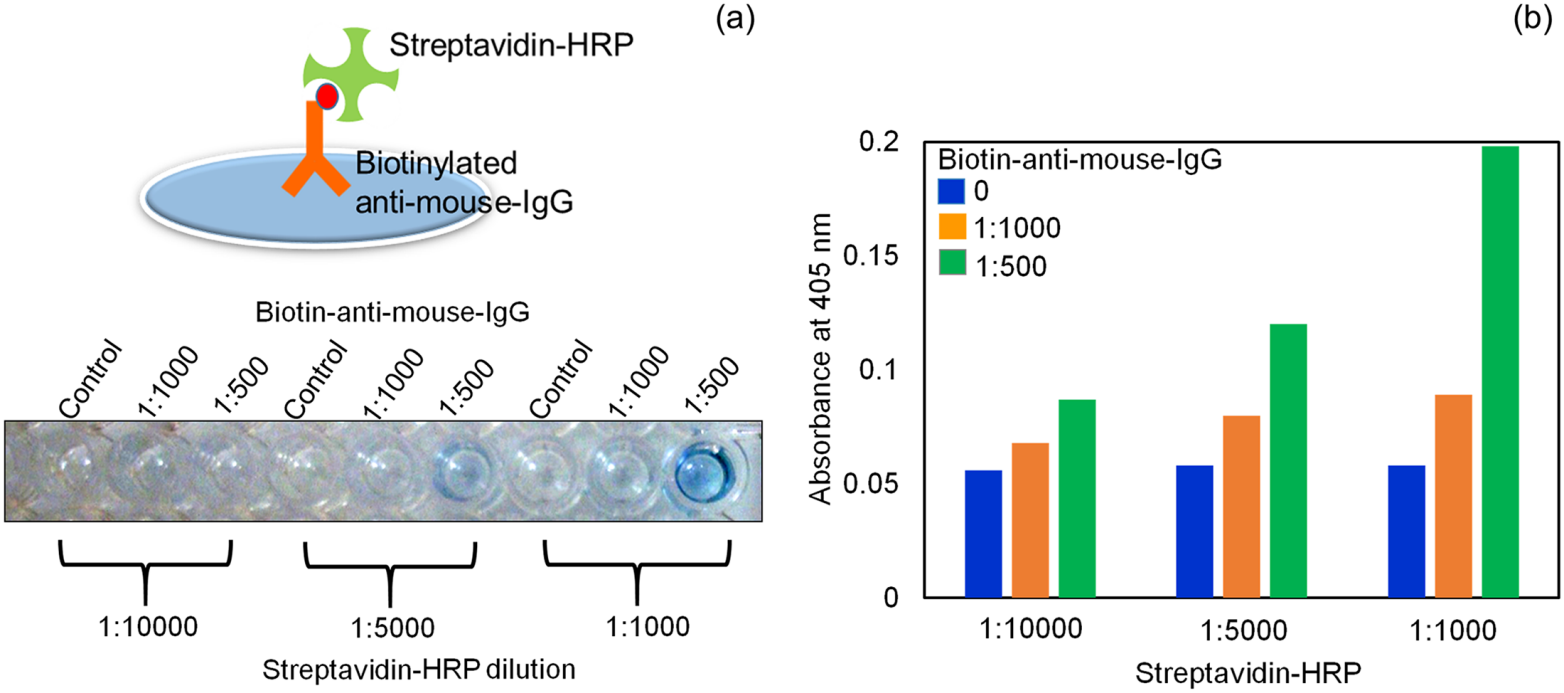
Optimization of biotinylated antibody and streptavidin-HRP. Two different dilutions of biotinylated antibody (1:500 and 1:1000) were coated onto ELISA plates. Different dilutions of streptavidin-HRP (1:1000, 1:5000, and 1:10000) were added and the interaction between biotinylated antibody and streptavidin-HRP was detected. (a) Color change in the solution after the addition of HRP substrate. (b) Graphical representation of the optimization.

Due to the importance of concentration of streptavidin-HRP and to attain the maximum sensitivity of the ELISA, we first determined the optimal dilution of biotinylated antibody and streptavidin-HRP. The combination of the 1:500 dilution of biotinylated antibody and the 1:1000 dilution of streptavidin-HRP resulted in the maximum OD (0.2), and no non-specific signal was detected in the control experiment (without biotinylated antibody) (Fig 2). Therefore, we used these two dilutions in the subsequent experiments.

### Addition of free biotin improved the interaction between biotinylated antibody and streptavidin-HRP

**Fig 3:**
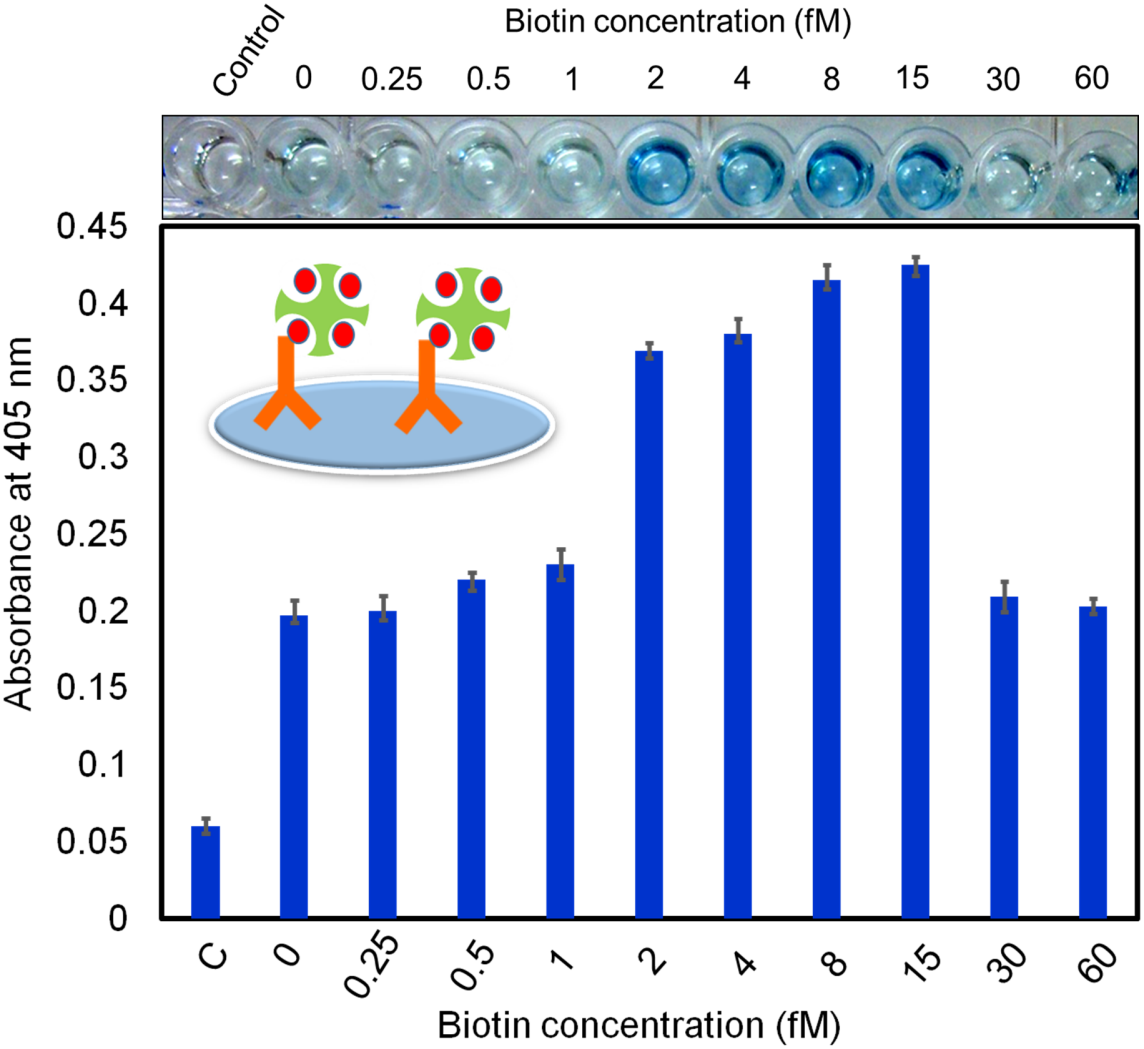
Addition of free biotin improved the interaction between biotinylated antibody and streptavidin-HRP. The 1:500 dilution of biotinylated antibody was detected by streptavidin-HRP mixed with different concentrations of free biotin (0 to 60 fM). Concentrations ranging from 2 to 15 nM of free biotin improved the detection, as indicated by the visible color change of the solution.

Because streptavidin has four binding sites for biotin, our goal was to block one or two of these binding sites with free biotin to provide more chances for streptavidin-HRP to bind with biotinylated antibody. We pre-mixed different concentrations of free biotin with streptavidin-HRP to determine the optimal concentration of free biotin. Biotin and streptavidin exhibit high affinity and a low dissociation constant [36], thus we expected saturation to occur quickly when we added the optimal concentration of biotin. If we added too much free biotin, it would occupy all four binding sites and not allow binding with the biotinylated antibody. To determine the optimal level, we carefully titrated the free biotin from 0 to 60 fM concentration. As shown in Fig 3, without biotin the OD was ~0.2 for the 1:500 dilution of anti-ESAT-6. As the free biotin concentration was increased, the OD also gradually increased. At 8 and 15 fM, the OD was ~0.425 for the 1:500 dilution of anti-ESAT-6 (i.e., almost 2.5 times higher than the OD for no biotin). This result illustrates that the addition of free biotin to streptavidin-HRP enhanced the binding of biotin and streptavidin. At the higher concentrations of biotin (30 and 60 fM), the OD was gradually decreased because more biotin binding sites on streptavidin-HRP were occupied by free biotin. The ELISA photographs clearly showed that with free biotin concentrations ranging from 2 to 15 fM the color of the solution changed to blue, which is indicative of higher OD (Fig 3). At 30 and 60 fM, however, the blue color is faint, indicating lower OD. These results show that 8 fM of free biotin is optimal for improving the interaction of biotinylated antibody and streptavidin-HRP.

### Interaction of anti-ESAT-6 and ESAT-6 in the absence of free biotin

**Fig 4:**
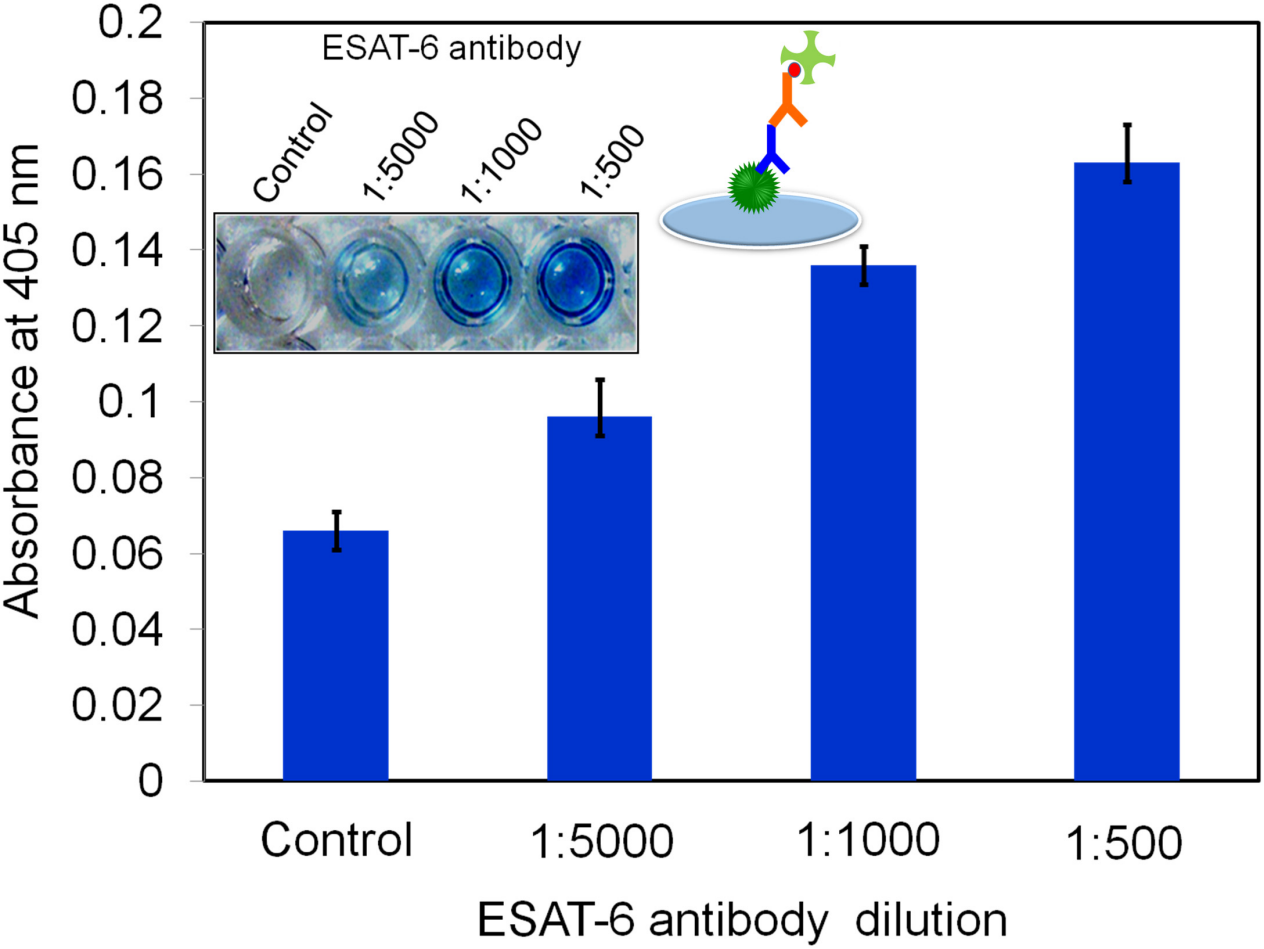
Optimization of ESAT-6 antibody. 100 nM of constant ESAT-6 coated on the ELISA well followed by adding the different dilutions (1:500, 1:1000 and 1:5000) of ESAT-6 antibody. And then detected by biotinylated antibody and streptavidin-HRP.1: 500 dilutions of ESAT-6 antibody showed the optimum dilution.

The next step was to determine the optimal dilution of anti-ESAT-6 for detecting ESAT-6. Different dilutions of anti-ESAT-6 were tested. The OD of the 1:500 dilution was higher (0.18) than that of the 1:5000 and 1:1000 dilutions for 200 nM of ESAT-6 (Fig 4). Photographs showed the dark blue color of the 1:500 dilution of anti-ESAT- 6. Thus, the 1:500 dilution of anti-ESAT- 6 was used in the subsequent experiments.

### Improved detection of ESAT-6 when free biotin was present

**Fig 5:**
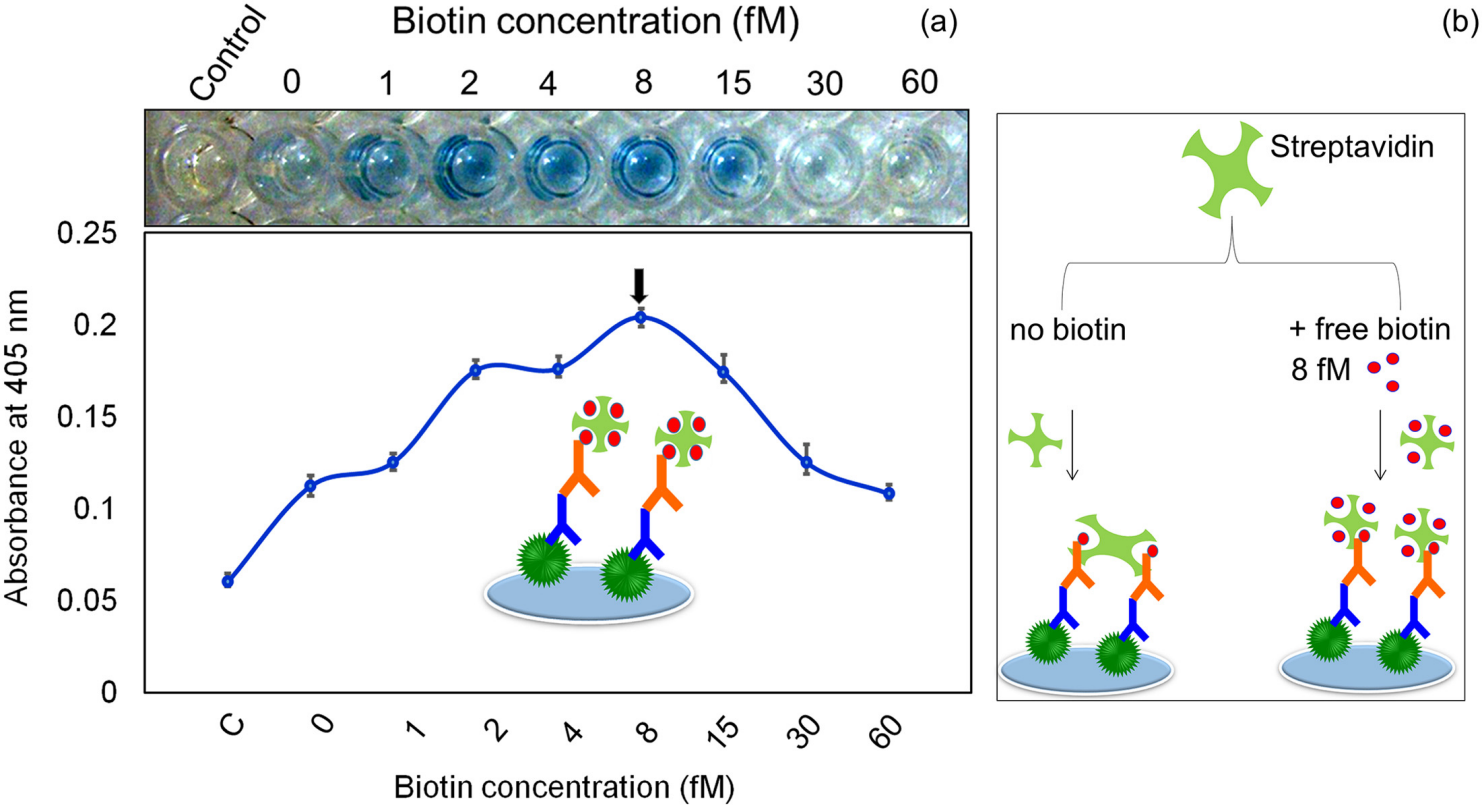
Addition of free biotin improved detection of ESAT-6. 100 nM of ESAT-6 were coated onto the ELISA plate, followed by ESAT-6 antibody and biotinylated anti-mouse-IgG. (a) Detection by streptavidin mixed with different concentrations of free biotin (0 to 60 fM) showed that 8 fM of free biotin resulted in the highest OD. (b) A model explains the enhanced sensitivity of streptavidin-HRP pretreated with a fixed amount (8 fM) of free biotin.

To determine the effect of free biotin on detection of ESAT-6, a constant concentration of ESAT-6 (100 nM) was detected using different concentrations of free biotin mixed with streptavidin-HRP. When no free biotin was present, the OD was ~0.12, and the OD was increased with increasing free biotin concentration (Fig 5a). The mixture of 8 fM of free biotin with streptavidin had the highest OD (~0.2), so we used this concentration to determine the LOD of ESAT-6. At concentrations higher than 8 fM, the OD was decreased. The ELISA photographs showed clear color changes in the presence of 1 to 15 fM of free biotin. It was proved that blocking some binding sites on streptavidin increased the binding of more streptavidin-HRP on the biotinylated antibody, ultimately there is an enhancement in the signal. To check the suitable concentration of free biotin, we titrated again with different biotin concentrations and found that 8 fM is more suitable to occupy some binding site(s) on streptavidin. When we increased further, it occupies all the biotin binding sites on streptavidin. With 8 fM, biotin binds only two or three binding sites on streptavidin, so that the remaining biotin binding sites on streptavidin-HRP are free to capture biotinylated antibody. A model is created that explains the enhanced sensitivity of streptavidin-HRP pretreated with a fixed amount of free biotin (Fig 5b).

### Limit of detection for ESAT-6

**Fig 6:**
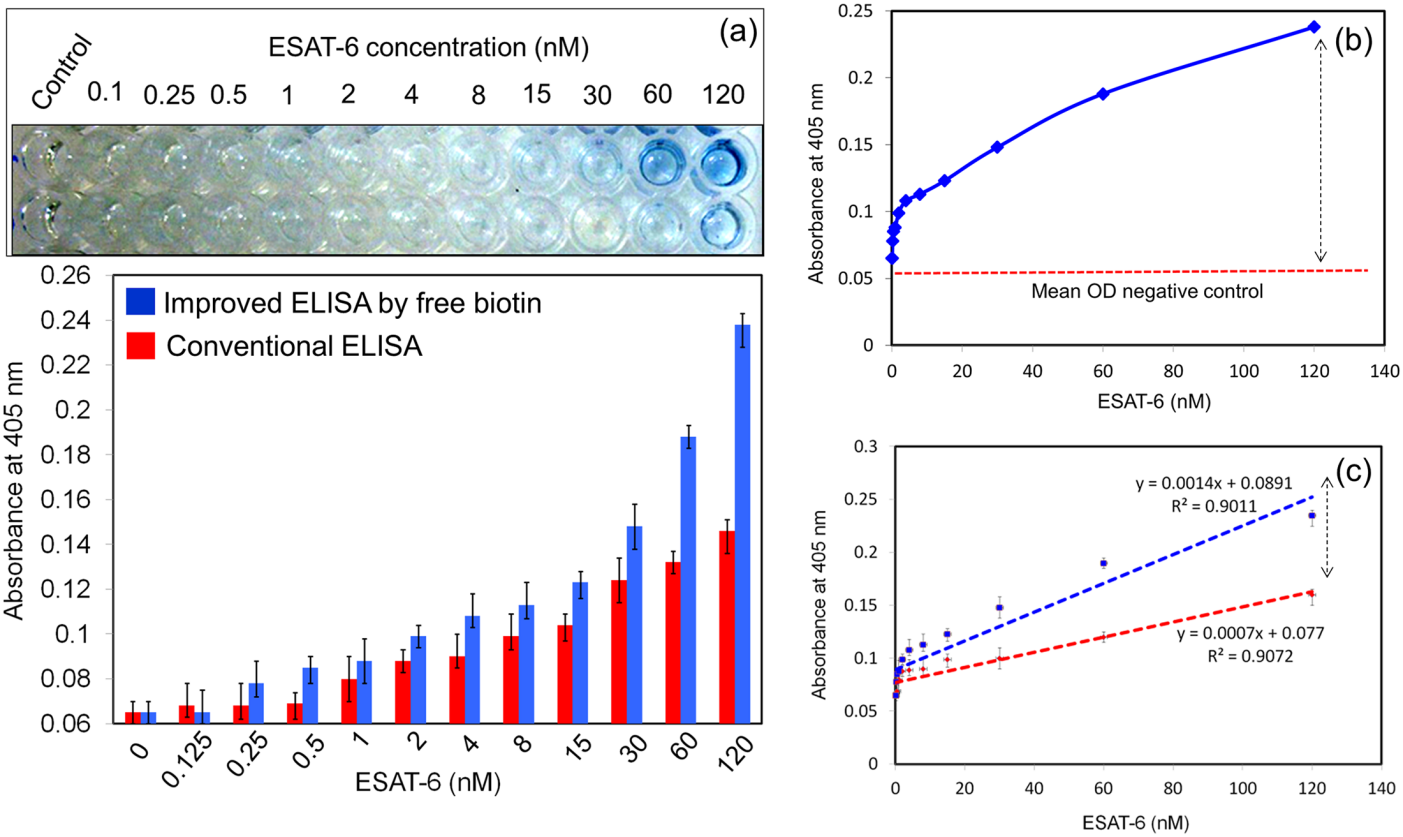
Limit of detection of ESAT-6. (a) ESAT-6 was titrated from 0 to 120 nM. LOD was compared between experiments with and without free biotin added to streptavidin-HRP. (b) Calculation of mean OD. (c) Statistical calculation on improved and conventional ELISA. R^2^ values were calculated and they indicate the significance.

To determine the LOD of the ELISA system with the pre-mixture of 8 fM free biotin and streptavidin-HRP, we titrated ESAT-6 from 0 to 120 nM. The LOD was found to be 250 pM (Fig 6a), which was a four-fold improvement over the system without free biotin (1 nM). Photographs were also clearly indicated the color changes which depends on the ESAT-6 concentrations. The LOD was calculated using statistical approaches by the method of blank ± 3 standard deviation (3σ). As shown in Fig 6b, the mean OD values were also calculated. R^2^ values of both conventional and improved ELISA with biotin showed the significant (Fig 6c). In fact, the OD was higher for all concentrations of ESAT- 6 when free biotin was present. In the control experiment, it didn’t show any non-specificity in both cases (with and without free biotin). This method is attested for many biological applications that involve the biotin and streptavidin interaction.

### Specific detection of ESAT-6: Spiking experiments

**Fig 7:**
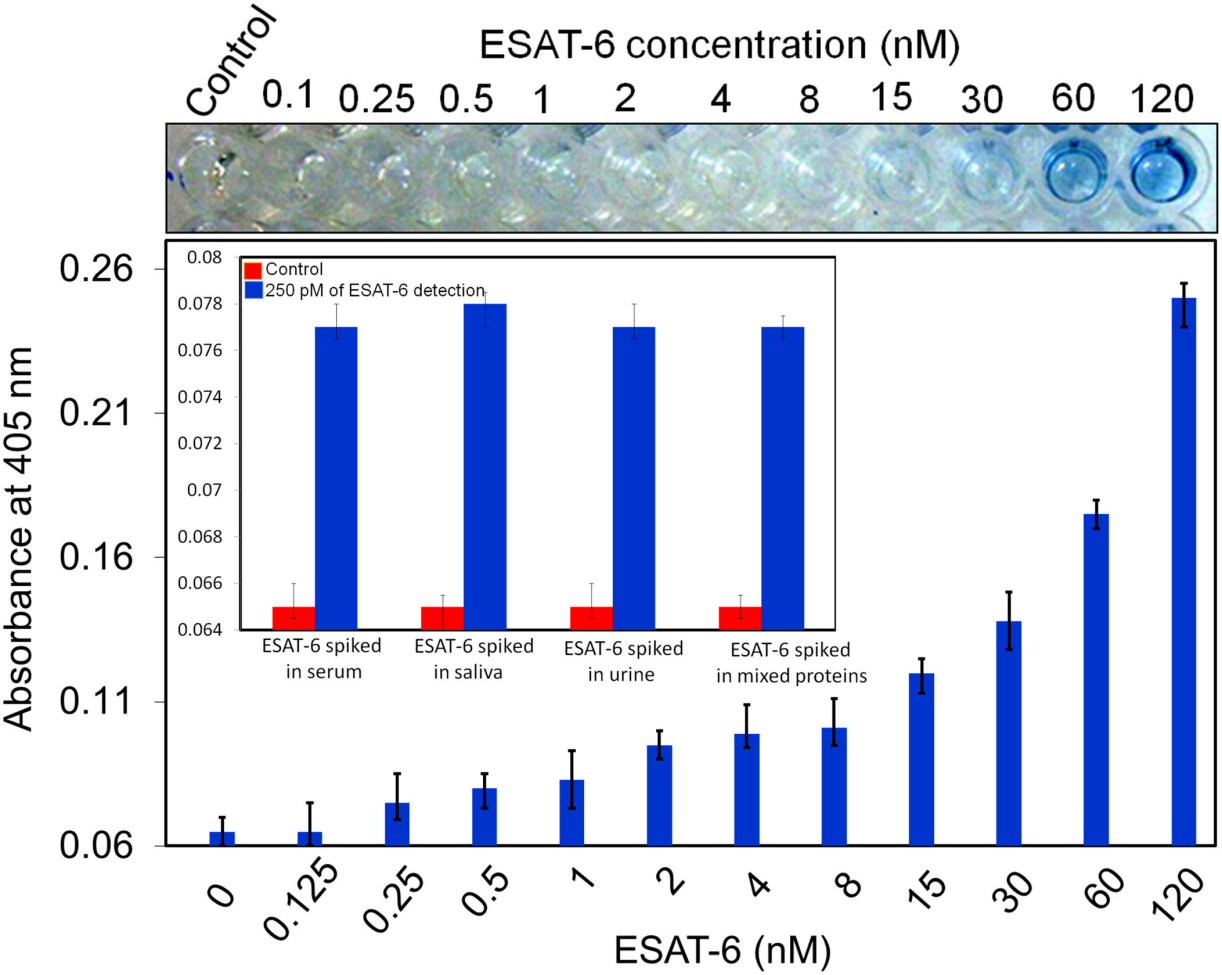
Specific detection of ESAT-6 in human serum. A 1:1000 dilution of human serum was spiked with different concentrations of ESAT-6. Specific detections were also confirmed by the spiking of 250 pM of ESAT-6 with human saliva, urine and mixture of other TB proteins (Fig inset).

Specificity is very important in a diagnosis system, particularly for mixed samples or crude samples such as serum or urine. The ability of our system to specifically detect ESAT-6 was tested by spiking a 1:1000 dilution of human serum with different concentrations of ESAT-6. Serum contains protein albumin at a concentration of 0.045 mg/mL [9]. Fig 7 shows that 120 nM of ESAT-6 was detected at a high OD value (0.24) and with a LOD of 250 pM (i.e., the same as that recorded in the absence of serum). We also evaluated spiking experiments with other crude media and to confirm the specificity at 250 pM of ESAT-6, we spiked in human saliva, urine and the mixture of TB proteins (ESAT-6, 16 kDa, Ag85B). As shown in Fig 7 (Fig inset), the result obtained in serum sample with 250 pM of ESAT-6 was also displayed in other media. These findings illustrated that diagnosis of ESAT-6 was not affected by the presence of human serum, saliva and urine and it was proved that ESAT-6 at low levels can be specifically detected by our developed ELISA strategy.

## Conclusion

ELISA is an immunoassay commonly used to detect different diseases using suitable antibodies. In this study, the ELISA method was improved to enhance the LOD for the given target using a competition-based strategy with biotin-streptavidin interaction. By blocking some of the biotin binding sites on streptavidin-HRP with free biotin, we enhanced the sensitivity of the system for detecting ESAT-6, an important protein secreted early during tuberculosis infection. The addition of pre-mixed free biotin (8 fM) with streptavidin-HRP improved the LOD four times relative to the system without free biotin (from 1 nM to 250 pM). Moreover, the LOD of ESAT-6 was also retained in the spiking experiments with human serum, saliva, urine and mixture of TB proteins. We could also reproduce the current strategy with other important targets and they are under progress. This competition-based strategy may also useful for other biotechnological applications in the future.
